# Parental, Prenatal, and Neonatal Associations With Ball Skills at Age 8 Using an Exposome Approach

**DOI:** 10.1177/0883073814530501

**Published:** 2014-05-14

**Authors:** Jean Golding, Steven Gregory, Yasmin Iles-Caven, Raghu Lingam, John M. Davis, Pauline Emmett, Colin D. Steer, Joseph R. Hibbeln

**Affiliations:** 1 Centre for Child and Adolescent Health, School of Social & Community Medicine, University of Bristol, Bristol, United Kingdom; 2 Department of Population Health, London School of Hygiene and Tropical Medicine, London, United Kingdom; 3 Department of Psychiatry, University of Illinois at Chicago, Chicago, IL, USA; 4 Section on Nutritional Neurosciences, National Institute of Alcohol Abuse & Alcoholism, National Institutes of Health, Bethesda, MD, USA

**Keywords:** ALSPAC, ball skills, motor coordination, maternal environmental background, exposome

## Abstract

There is little consistency in the literature concerning factors that influence motor coordination in children. A hypothesis-free “exposome” approach was used with 7359 children using longitudinal information covering 3 generations in regard to throwing a ball accurately at age 7 years. The analyses showed an independent robust negative association with mother’s unhappiness in her midchildhood (6-11 years). No such association was present for study fathers. The offspring of parents who described themselves as having poor eyesight had poorer ability. This hypothesis-free approach has identified a strong negative association with an unhappy childhood. Future studies of this cohort will be used to determine whether the mechanism is manifest through differing parenting skills, or a biological mechanism reflecting epigenetic effects.

Child development is fundamental to the life chances of young people as they grow to adulthood.^1,2^ Motor ability is, in particular, considered by children and adolescents to be very important, and those with poor abilities are at increased risk of being bullied and having poor self-esteem.^3^ Nevertheless, the topic has received relatively little attention compared with cognitive development. In reviewing the literature, we have shown that, of the few studies concerning etiology, there is little consistency in either the measurement strategy, or the ages at which the individuals are measured, and consequently little consensus as to causal influences.^4^

Although it is well documented that heredity plays an important role in the individual’s motor skills,^5^ there is also evidence of a role for the environment, although little consistency as to which aspects of the environment are important. Among the features that show some suggestions of a causal role (whether positive or negative) in the literature, the majority relate to the prenatal period.^4^ They include (*a*) prenatal exposures to lead, mercury, and arsenic; (*b*) deficiencies of iron and iodine; (*c*) exposure to other persistent chemicals such as PCBs and various pesticides; (*d*) social drug exposure such as excessive alcohol, cigarette smoking, and hard drugs such as cocaine; (*e*) prenatal dietary factors such as seafood, and postnatal exposures such as breast feeding had been shown to be of benefit, and assumed to be due to omega-3 fatty acid ingestion^6^; (*f*) prenatal exposure to medications such as selective serotonin reuptake inhibitors; (*g*) fetal asphyxia and neonatal complications.

In spite of the relatively large number of studies reviewed, there was little consistency in results. In part this is likely to be because (*a*) there was no consistency in the tests used, or of the facets of motor skills considered, and (*b*) there is good evidence in longitudinal studies that there is a lack of consistency in development of motor ability over time in young children, such that children who score poorly at one age are not necessarily those who score poorly a few months or years later.^7,8^

In an attempt to counteract the problems of consistency over time, and in regard to the tests used, we concentrate on an age (7 years) by which the motor abilities of the children are likely to have become more settled. We report here on one test adapted from the Movement Assessment Battery for Children,^9^ that of throwing a ball accurately. This is claimed to be an excellent way of testing a child’s gross motor coordination (and to a lesser degree, fine motor coordination).^10^ Because there have been no studies that have looked at a broad range of environmental influences, we have taken a hypothesis-free approach to identify associations that may indicate ways in which the environment prior to the child’s birth may influence the development of ball skills.

We used a well-characterized cohort of children for whom data on pregnancy and the postnatal environment had been collected prospectively (the Avon Longitudinal Study of Parents and Children [ALSPAC]). This study utilizes information on the pregnancy collected prospectively as well as data collected retrospectively concerning the preconception period from the time of the parents’ births as well as of details of their own parents.^11–13^

## Methods

### Study Sample

The data used in these analyses were collected as part of the Avon Longitudinal Study of Parents and Children, which was designed to assess the ways in which the environment interacts with the genotype to influence health and development.^11^ Pregnant women, resident in the study area in southwest England with an expected date of delivery between April 1, 1991, and December 31, 1992, were invited to take part. About 80% of the eligible population did so.^12,13^ Data were obtained from the parents during pregnancy and subsequently, biological samples were collected from the mothers during pregnancy and from the children at a variety of time points. In addition, the study children were examined at various ages. Full details appear on the Avon Longitudinal Study of Parents and Children website http://bristol.ac.uk/alspac/.

### Ball Skills

**Figure 1.**
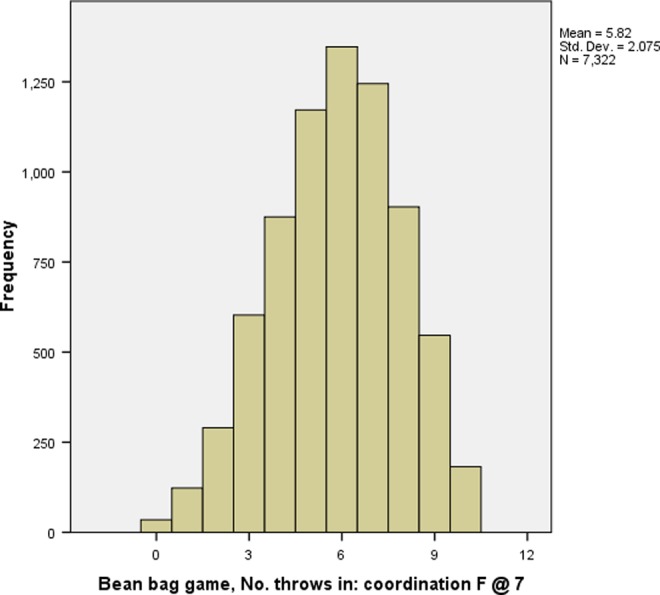
Distribution of the frequency with which the children threw the ball into the box (7322 children tested).

The Avon Longitudinal Study of Parents and Children Coordination Test was derived from subtests of the Movement Assessment Battery for Children (the Movement ABC or MABC)^9^ and used when the children were approximately 7½ years of age. It was carried out in rooms adapted for the study and conducted by trained examiners from the Avon Longitudinal Study of Parents and Children. Parents accompanied children but were not allowed to help them. From the ball skills section of the Movement Assessment Battery for Children, the bean bag subtest was conducted. This involved the child attempting to throw a bean bag (underarm) into a box, while standing behind a line at a distance of 6 feet from the box. During the demonstration and explanation, the tester emphasized to the child that he or she should use only one-handed, underarm throws and remain behind the line for each throw, standing in a good position for throwing. The children were given 5 practice throws where they were able to change hands but were encouraged to choose their preferred hand for the main trial. Any procedural errors made during the practice were corrected and the children were reminded of, or redemonstrated, the correct procedure. Out of 10 throws, the number to successfully land in the box was recorded. The resulting data were approximately normally distributed (Figure 1) and had a mean of 5.82 and standard deviation 2.08.

### Measurement of Exposures

The women and their partners were sent 4 and 2 questionnaires, respectively, during the study pregnancy. These elicited information on their parents, their early childhood, adolescence, and adulthood. The study website contains details of all the data that are available through a fully searchable data dictionary: http://www.bris.ac.uk/alspac/researchers/data-access/data-dictionary/.

The data analyzed for this study comprises information collected up until the time of birth of the study child and at the end of the first month of the child’s life. As well as details collected from the questionnaires, we include details from the obstetric records (where available) and results of assays of the mothers’ biological samples. A list of the sources and coding of the 1755 items considered up until the neonatal period is available from the corresponding author and summarized in this study. The variables considered included the following for which there is some evidence of an effect on motor development: maternal blood levels of lead and mercury, anemia, exposure to pesticides, alcohol, cigarette smoking, hard drugs, medications for different conditions including depression, details of dietary intake including seafood, various measures of fetal asphyxia including Apgar scores, neonatal morbidity, and breast feeding.^4^

### The Strategy Using an “Exposome” Approach

Although there is considerable evidence to suggest that environmental factors are involved in the development of many childhood and adult outcomes, it is largely agreed that events or conditions in the prenatal or infant period may have a key effect on many pathways. Increasingly attention is being concentrated on even earlier time frames, with the possible influence of parental childhood, infancy, and fetal life.^14^ In this study, we assess the ways in which the grandparents, the parent’s birth, childhood, adolescence and early adulthood, the environment prior to conception, during pregnancy, labor, delivery, and the neonatal period may influence the child’s motor ability as demonstrated using a test of ball skills.

### Statistical Approach

**Figure 2.**
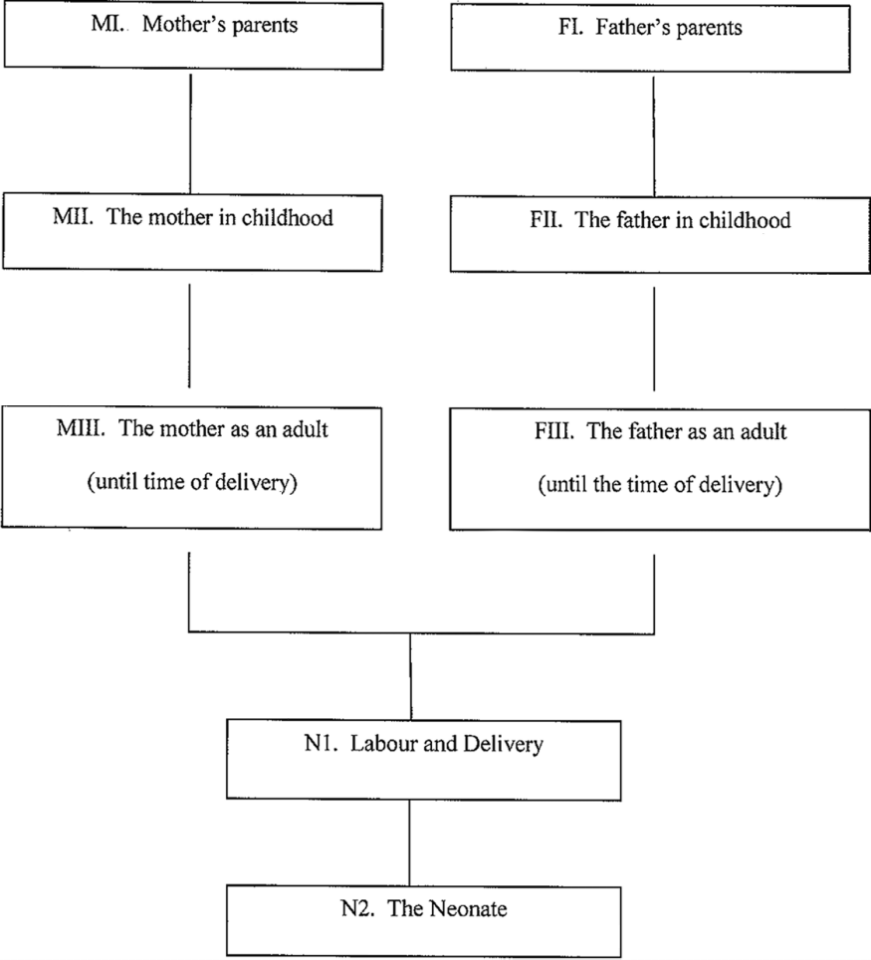
Diagram showing the different types of variables considered in the analysis.

We treat the measures using an “exposome” analysis. This is similar to the approach taken in genomewide association studies, being hypothesis free. Rather than testing already formulated hypotheses, a genomewide association study examines associations with as many as 2 million genetic markers for predetermined statistical significance levels and then attempts to replicate the findings in other studies. In general, no adjustment is made for confounders. In the present study, which is a hypothesis generator in regard to the environmental factors, we first assess the relationship of ball skills with each environmental variable categorizing the *P* values <.05, <.01, and <.001 divided into the times and route of exposure (Figure 2). We then assess whether the numbers of variables identified in this way occur more often than expected by chance. In particular, we look for unexpected patterns of association.

In order to ensure that we do not suffer from type II errors, we do not correct for multiple testing at this stage, although we do concentrate first on the variables that are univariably associated at the 1% rather than the 5% level. In order to look for pattern, the environmental variables are divided and considered in fairly logical groupings. For each set of analyses of the different groupings, we use backward linear regression. We then use a logical set of sequences of the independent variables from each group and combine the variables into a final model.

We also apply a sensitivity analysis. In this case, we use the same set of initial variables and carry out a similar set of backward analyses, but this time the outcome variable concerns the odds ratio associated with poor ball skills defined as lower than 1 standard deviation from the mean (ie, <4 accurate throws).

## Results

Overall, 7841 children attended the 7-year assessments. As can be seen from Table 1, there were biases in those attending compared with those who did not in regard to maternal age, education level, housing tenure, and ethnic background; however, there were no statistically significant differences in gestation or birth weight. Of those attending, 7359 (94%) completed the ball skills test—the reasons for noncompletion were mainly related to organizational failures and thus were not specifically related to social characteristics.

**Table 1.** Differences in Characteristics of Attendees at 7-Year Assessments Compared to Nonattendees.

	Attendees (n = 7841), n (%)	Nonattendees alive at 1 y (n = 6130), n (%)
Gender		
Male	3988 (50.9)	3226 (52.6)
Female	3853 (49.1)	2902 (47.4)
Maternal education		
<O Level	1650 (21.9)	2074 (42.5)
O Level	2673 (35.5)	1627 (33.4)
A Level or higher	3217 (42.7)	1174 (24.1)
Maternal age		
<20	253 (3.2)	749 (12.2)
20-24	970 (12.4)	1362 (22.2)
25-29	3099 (39.5)	2300 (37.5)
30-34	2560 (32.6)	1296 (21.1)
≥35	959 (12.2)	423 (6.9)
Housing tenure		
Owner-occupied	6278 (82.6)	3284 (60.6)
Public rented	729 (9.6)	1348 (24.9)
Other rented	591 (7.8)	791 (14.6)
Ethnicity of child		
White	7122 (95.9)	4350 (93.4)
Non-white	301 (4.1)	308 (6.6)
Maternal age, M (SD)	29.05 (4.6)	26.65 (5.1)
Birthweight, M (SD)	3417 (559)	3358 (562)
Gestation, M (SD)	39.44 (1.9)	39.41 (1.9)

Abbreviations: M, mean; SD, standard deviation.

The initial comparison of the different groups of variables considered in this study is shown in Table 2. Overall 11.9%, 4.3%, and 1.2% of the variables were associated with mean ball skills at the 5%, 1%, and 0.1% levels, respectively. Thus there were twice as many as would be expected by chance at the 5% level, 4 times more at the 1% and 12 times more at the 0.1% level; each of these statistics was significantly in excess. However, the excess in regard to the groups of variables was not uniform. There was no evidence of an excess of associations for MI or FI (the study children’s grandparents), FII (the father’s childhood), or NI (characteristics of labor and delivery). We do not consider variables in these groups further here, although the significant associations are described in the Supplementary Material. We have analyzed each of the other groups of variables further.

**Table 2.** Number of Variables (%) Associated Univariably With Ball Skills Score.

		Number of variables (%)		
Group of variables	n^a^	*P* < .05	*P* < .01	*P* < .001
MI. Mother’s parents	51	2 (3.9)	0	0
FI. Father’s parents	49	2 (4.1)	1 (2.0)	0
MII. Mother’s childhood	184	23 (12.5)	11 (6.0)	4 (2.2)
FII. Father’s childhood	106	7 (6.6)	2 (1.9)	1 (0.9)
MIII. Mother as an adult^b^	926	117 (12.6)	37 (4.0)	7 (0.8)
FIII. Father as an adult^b^	268	42 (15.7)	16 (6.0)	3 (1.1)
N1. Characteristics of labor and delivery	84	2 (2.4)	0	0
N2. The neonate^c^	87	13 (14.9)	9 (10.3)	6 (6.8)
Total	1755	208 (11.9)	76 (4.3)	21 (1.2)

^a^Number of variables considered.

^b^Until the time the baby was born.

^c^The first 4 wk after delivery.

### MII. The Mother’s Childhood

Of the 184 measurements of conditions and events during the mother’s childhood, 11 were significant at the 1% level (6 times more than expected), and 4 at the 0.1% level (22 times more than expected); details are listed in the Supplementary Material. Almost all the variables involved were related to descriptions of a traumatic childhood, with better ball skills for women who perceived their childhood and adolescence as happy, their mother as caring, and the mother and home as stable. Mothers who had been physically or emotionally abused in childhood, and those who had spent time in a Children’s Home, were more likely to have children with poor ball skills. Curiously, the heavier the mother at birth and the presence of a stepsister during the mother’s adolescence were associated with better ball skills in the study child.

The factors outlined here were offered to a backwards stepwise analysis, with the exception of the mother’s birth weight; this was omitted because of the large amount of missing data, and some doubt as to the accuracy of this variable. Only 1 variable remained as independently associated with a reduction in ball skills in their children in this analysis: whether the mother had stayed in a Children’s Home at any point (*P* = .018); conversely, the degree of happiness in childhood when aged 6 to 11 (*P* < .0001) and the presence of a stepsister in the household when the mother was aged 12 to 15 (*P* < .001) were associated independently with better skills in the children (Supplementary Table 1A). The variables not independently associated were subsumed by the variable concerning the mother’s recollection of her happiness or lack of it in midchildhood—this particularly applied to the stability of the home and whether the mother had been abused.

### MIII. The Mother as an Adult

The factors associated with ball skills at the 1% level were considered in 3 groups: her home environment, her social and health characteristics, and her diet during pregnancy. These we analyzed as separate subgroups before combining them.

Features of the home environment that were associated with better ball skills of her offspring included the length of time the mother had lived in Avon (the study area), the number of rooms that had been wall-papered in the year prior to pregnancy, the frequency with which windows were kept open in the summer, the frequency with which aerosols, an iron, or electric hair appliances were used and the number of children in the household; conversely the following factors had negative associations with ball skills: the number of household moves in the previous 5 years, the degree of damp in the home, whether cats were in the home or dogs were seen as pests in the vicinity, and the more polluted the neighborhood was. Multiple stepwise regression of this group of variables resulted in just 4 variables dropping out—the time the mother had lived in Avon, the number of household moves in the past 5 years, the frequency with which she used an electric iron, and the levels of pollution in the neighborhood (Supplementary Table 1B). The analysis resulted in an increase in the regression coefficients for the negative effect of cats in the home (from –0.14 to –0.17), and for the positive effect of the number of children in the home (from 0.08 to 0.12).

In regard to social and health variables, the following were associated univariably with increased ball skills: having had a head injury, length of time on the contraceptive pill, having had diarrhea in pregnancy, her partner having children that do not live with her, the number of living children the mother has, and whether she claims that her religion is Church of England. In regard to negative associations, these include whether she had poor vision, had had an x ray of the leg or foot in pregnancy, and whether she had had an x ray in the last months of pregnancy. In addition, there were reduced levels of skill in women who were worried over being burgled or felt they were discriminated against because of the way they dressed. There were negative associations also with the frequency with which they attended a place of worship, and whether they obtained help from members of their religion. Unexpectedly, the higher the level of education the mother had achieved, the lower the skill that the child had with a ball. Multiple regression of this group of variables resulted in 6 being dropped—the mother’s educational level, number of living children, history of diarrhea, and x rays in pregnancy and whether she had help from others in her religious group (Supplementary Table 1C).

Finally, there were a number of unadjusted associations with the mother’s diet in pregnancy. These included positive effects on the child’s ball skills with the frequency with which she had milk in tea, used solid fat (eg, butter, lard) for frying, frequency with which she ate poultry, or potato chips (known as “crisps” in the United Kingdom), had alcoholic binges in midpregnancy, or went on a diet to lose weight. There were negative associations with the frequency with which she ate pulses, drank herbal teas, and with whether she was a vegetarian. Only 2 variables dropped out when this group were analyzed together: the eating of pulses and the vegetarian status of the woman (Supplementary Table 1D).

### The Father’s Contribution

There were 15 variables related to the study father prior to birth that were univariably associated at the 1% level with the ball skills of the child. These involved positive associations with the frequency with which he ate meat products such as pies, length of time he had lived in Avon, and whether he used a deodorant, but negative associations with being a vegetarian, personality measures of fragile inner self and interpersonal sensitivity, the frequency with which he attended a place of worship, obtained help from a leader of religion and from other members of his religion, sight in left and right eye and a summary measure of poor sight, low temperature in bedrooms in winter, exposure to radiation, and anxiety concerning being burgled (Supplementary Tables 1G and 1H). The model using backwards regression on these variables (Supplementary Table 1J) showed that just 5 variables remained in the model—these were dominated by the summary variable “any sight problem” (*P* < .001). The other variables remaining at the 5% level were the frequency of eating meat pies, fragile inner self personality, use of deodorant, and exposure to radiation.

### The Fetus and Neonate

As predicted, boys had much better ball skills than girls on average. Although there was an association with birth weight, birth length, and head circumference (all positive), this was largely due to the sex difference in skills and to the fact that babies delivered at term had substantially better ball skills subsequently than those born preterm. Nevertheless, there was still a marginally independent positive effect with birth weight (*P* = .033) (Supplementary Table 1E).

Other features of the neonate were available on smaller numbers (Supplementary Table 1F), and showed unadjusted negative associations with the resuscitation of the baby, especially if performed using intubation and IPPV, and whether the baby was transferred for special care. Allowance for gestation, birth weight, and sex showed only resuscitation to have an independent effect (*b* = –0.18, 95% CI = –0.32, –0.03; *P* = .015).

### The Final Models

Combination of all independently associated variables from the 4 regression analyses concerning the mother (ie, her childhood, her home environment, her social and health characteristics, and her diet during pregnancy) resulted in 18 variables remaining (Table 3). Those relating to her history of staying in a children’s home, having milk in tea, drinking herbal tea, using aerosols, partner’s children living elsewhere, and describing herself as Church of England were all eliminated. The resulting model included just 2 variables from childhood, but they were both significant at the 1% level: happiness in midchildhood was the most significant effect in the model (*P* < .001). The other 5 variables significant at the 1% level included 2 related to diet (hard fat used for frying; frequency of dieting), 2 to animal proximity (cats in the home, dogs as pests), and 1 social factor (frequency mother attends place of worship). One other noteworthy variable, although significant at only 5%, that had a strong negative effect was poor maternal vision.

**Table 3.** Independent Features of the Mother During Her Childhood and Up Until the Time of Birth of the Study Child Related to the Child’s Skill at Throwing a Ball Accurately: Results of Backward Stepwise Regression on Score of Successful Ball Throwing.^a^

Variable	n	*P*	b (95% CI)
Happiness in early childhood: 6-11	5514	<.001***	0.14 (0.07, 0.22)
Stepsister present: 12-15	5514	.004**	0.70 (0.22, 1.17)
Frequency of eating poultry in pregnancy	5514	.012*	0.10 (0.02, 0.18)
Butter/lard/fat used for frying in pregnancy	5514	.005**	0.19 (0.06, 0.32)
Frequency of eating potato chips in pregnancy	5514	.012*	0.07 (0.01, 0.12)
Number of times mother has dieted	5514	.009**	0.07 (0.02, 0.11)
Frequency of binge drinking midpregnancy	5514	.040*	0.09 (0.00, 0.17)
Frequency windows open on summer day	5514	.011*	0.12 (0.03, 0.20)
Cats in the home	5514	.008**	–0.16 (–0.27, –0.04)
Dogs as pests	5514	.004**	–0.14 (–0.23, –0.04)
Level of dampness/condensation in home	5514	.040*	–0.10 (–0.19, 0.00)
Number of areas wallpapered in last year	5514	.012*	0.07 (0.01, 0.12)
Frequency of electric hair appliance use	5514	.010**	0.06 (0.01, 0.10)
Number of children in household	5514	.010**	0.09 (0.02, 0.16)
Had a head injury	5514	.035*	0.15 (0.01, 0.29)
Poor vision	5514	.015*	–0.28 (–0.51, –0.06)
Frequency mother attends place of worship	5514	.002**	–0.09 (–0.15, –0.04)
Feels discriminated against because of dress	5514	.039*	–0.38 (–0.74, –0.02)

Abbreviations: b = regression coefficient; CI = confidence interval.

^a^Total n = 5514, overall *R* ^2^ = 2.53%.

*P < .05; **P < .01; ***P < .001.

Next the variables relating to the neonate were added to the model in Table 3 to assess whether the mechanism by which any of the maternal variables had their effect was through the birth characteristics of the infant. The resulting analysis indicated that this may have explained the relations with binge drinking, and the feeling of discrimination because of dress, but the changes in effect size were small (Table 4).

**Table 4.** Independent Features of the Mother and Characteristics of the Child at Birth Related to the Child’s Skill at Throwing a Ball Accurately: Results of Backwards Stepwise Regression on Score of Successful Ball Throwing.^a^

Variable	n	*P*	b (95% CI)
Sex: girl vs boy	5621	<.0001****	–0.74 (–0.85, –0.64)
Gestation at delivery^b^	5621	<.0001****	0.44 (0.22, 0.66)
Happiness in early childhood: 6-11	5621	<.0001****	0.15 (0.07, 0.22)
Stepsister present: 12-15	5621	.002**	0.73 (0.28, 1.19)
Frequency of eating poultry in pregnancy	5621	.015*	0.09 (0.02, 0.17)
Butter/lard/fat used for frying in pregnancy	5621	.001**	0.21 (0.08, 0.35)
Frequency of eating potato chips in pregnancy	5621	.003**	0.08 (0.03, 0.13)
Number of times mother has dieted	5621	.010*	0.06 (0.01, 0.11)
Frequency windows open on summer day	5621	.016*	0.11 (0.02, 0.19)
Cats in the home	5621	.011*	–0.15 (–0.26, –0.03)
Dogs as pests	5621	.003**	–0.14 (–0.23, –0.05)
Level of damp/condensation in home	5621	.043*	–0.09 (–0.19, 0.00)
Number of areas wallpapered in last year	5621	.014*	0.06 (0.01, 0.11)
Frequency of electric hair appliance use	5621	.010*	0.06 (0.01, 0.10)
Number of children in household	5621	.016*	0.08 (0.02, 0.15)
Had a head injury	5621	.024*	0.16 (0.02, 0.30)
Poor vision	5621	.025*	–0.25 (–0.47, –0.03)
Frequency mother attends place of worship	5621	.001**	–0.10 (–0.15, –0.04)

Abbreviations: b = regression coefficient; CI = confidence interval.

*P < .05; **P < .01; ****P < .0001.

^a^Total n = 5621, overall *R* ^2^ = 5.81%.

^b^Coded in 3 categories: < 32, 32-36, ≥ 37 weeks.

Finally we integrated the variables shown to be associated with the partner. This resulted in a reduction in the numbers considered from 5621 to 4017, with a consequent reduction in power. Nevertheless, there were 15 variables that were independently associated with ball skills, those that were significant at the 1% level were the father having a sight problem, the sex of the child, and gestation at which the child was born, the mother’s happiness in midchildhood, presence of a stepsister during the mother’s adolescence, solid fat used for frying the food she ate, number of times mother has dieted, whether dogs are pests locally, and the frequency with which the mother attends a place of worship (Supplementary Table 2). However, addition of the variables concerning the father resulted in only a marginal increase in the *R* ^2^ from 5.81% to 5.90%. Consequently, since the numbers involved are much larger without the additional paternal variables, we consider Table 4 to be the Final Model, but keep in mind the fact that there was a pronounced effect with paternal sight problems.

### Sensitivity Analyses

Of the 18 variables in the logistic regression model that excluded variables relating to the father (Table 4), all were univariably associated with the risk of poor ball skills. Stepwise logistic regression resulted in 8 demonstrating independent associations. In relation to the child, these included girls having worse abilities (*P* < .0001), and those born at term more likely to have better skills (*P* = .022). In regard to the mother, the following were associated with better skills: maternal happiness level in her midchildhood (*P* = .002), having had a history of head injury (*P* = .008), frequency of eating potato chips (*P* = .009), and the number of areas wallpapered in the home in the year prior to midpregnancy (*P* = .036); conversely, having a cat in the home (*P* = .002) and dogs as pests in the area (*P* = .001) were associated with increased likelihood of poor abilities (Supplementary Table 3). Thus, the evidence for these variables being associated with ball skills is enhanced, though many of them could still be associated by chance.

Offering the paternal variables resulted in the association with maternal childhood happiness becoming more statistically significant (*P* < .001). Similar to the linear regression analysis, there was an association with paternal sight problems such that his offspring were more likely to have poor ability (*P* = .012), and the frequency of the father eating meat products was associated with increased ability (*P* = .013).

## Discussion

We have used a test of accuracy in throwing a ball as a measure of motor coordination using a large population-based study of 7-year-old children. This test has the advantage of giving a normally distributed set of results with no ceiling effects at this age. These data were used in association with data collected during pregnancy and immediately after the child’s birth to assess which factors in the parental and neonatal background might be associated with this ability. This is the first study using an “exposome” approach to examine the possible associations with a large number of environmental features. We first assessed the unadjusted associations with mean test score and showed that certain groups of variables were particularly associated: viz the mother’s childhood, the mother as an adult, the father in adulthood, and the neonate. We did not find more significant associations than expected by chance with details of the grandparents on either side nor with the father’s childhood; therefore, we did not assess these areas further. For areas with high numbers of variables associated with ball skills we used a structured set of backward stepwise linear regression analyses to demonstrate the findings that were independently associated with ball throwing ability. We found a number of such relations, which we discuss below.

Our initial hypotheses had been based on the literature concerning motor development in general.^4^ Prenatal exposures to toxic metals and other chemicals, deficiencies of essential nutrients, social drugs such as alcohol and nicotine, and medications such as selective serotonin reuptake inhibitors were all predicted to have harmful effects; in addition, prenatal dietary components such as seafood, and postnatal exposures such as breast feeding to have beneficial effects. Poor social circumstances were reported to be associated with reduced abilities. Of these, we assessed and found no effect with blood levels of lead, mercury, cadmium, selenium, medications taken, smoking history of the mother and her partner, environmental tobacco smoke exposure, regular alcohol intake, seafood intake, or history of being breast-fed. We did find an association with binge drinking in midpregnancy that appeared to be due to an association with preterm delivery. Of the birth histories hypothesized from our literature review, gestation at delivery was retained. There was no association with Apgar score, but that with resuscitation was retained though at much reduced significance.

The aim of an exposome analysis such as this is to identify previously unsuspected associations; the technique is thus both hypothesis testing and hypothesis generating. In this study, one of the major findings concerned the mother’s traumatic childhood. The unadjusted analyses showed that the offspring of mothers who had been abused physically or emotionally, those who had rated their own mothers as uncaring and/or unstable, and who had spent some time in a Children’s Home were more likely to have offspring with poor ball skills. These aspects of their childhood were summarized in a variable that they used to rate their happiness during childhood; this rating was positively related to ball skills. This was robust to adjustment and continued to show strong effects throughout, with the more happy the mother had been in mid-childhood, the better the ball skills of her child (*P* < .001), or conversely the more unhappy the worse the study child’s ability with a ball. The key question concerns whether this could be causal. The association is unlikely to be due to associations with unmeasured factors (eg, a social patterning), as we would expect similar associations with the fathers’ background if that were true. There is increasing evidence that stress in early childhood can have a long-term effect on the brain of the individual,^15^ and consequently this may have affected the mother’s own motor abilities. Maternal history of childhood abuse has also been associated with the function of the hypothalamic-pituitary-adrenal (HPA) axis in both the mother and the infant,^16^ though how that might affect subsequent motor skills is for conjecture as yet. One possible explanation is that of an epigenetic effect on the mother, and thence to her child, thus affecting her child’s motor coordination.

It is likely that parental abilities have a major influence on the child’s motor development. Dusenberry^17^ showed that specific training, even for a short time period resulted in improved ball skills, and parental involvement playing with a ball with the child is likely to result in improved skills in children of all innate abilities. This may be the explanation for the negative association with factors that possibly indicate poor skills in the parents such as poor eyesight, and features of the environment that restrict relaxed access to the outside areas (for practicing ball skills) such as uncontrolled dogs in the neighborhood.

Although Gardener^10^ stated that throwing balls in a basket is an excellent way of testing gross motor coordination (and to a lesser degree, fine motor coordination), using the Avon Longitudinal Study of Parents and Children cohort, Howard^18^ has demonstrated a link between ability with ball skills and a measure of binocular vision. She and others have shown in the Avon Longitudinal Study of Parents and Children that binocular vision was strongly and beneficially associated with duration of breast feeding and maternal intake of seafood in pregnancy.^19^ Had we found an association between these 2 dietary intakes and enhanced ability with ball throwing skill, we would have concluded that the explanation concerned an association with binocular vision. However, we did not find any associations with foods that would have increased omega-3 levels in the mother during pregnancy, oily fish for example; on the contrary, we found positive associations with the use of solid fat for frying and the frequency with which the mother ate potato chips.

There were associations in these data that reflected the literature. Watson and Kimura^20^ stated that, in general, males excel on psychometric tests of spatial ability, whereas females enjoy an advantage on tests of verbal and fine motor ability. We found the gender difference to be the most significant in our model (*P* < .0001) with boys making more accurate throws than girls. Similarly there was a strong negative association with preterm delivery, reflecting a study of 12- to 13-year-olds in Australia tested using the Movement Assessment Battery for Children: this showed a strong linear association with their gestational age at birth for the total score, but that when divided into its subcategories, the association was predominantly due to an association with ball skills rather than manual dexterity.^21^

Curiously we found no independent associations with social conditions either in the parents’ own childhood or during the current pregnancy. However, in unadjusted analyses, the children of the more educated mothers had the lowest abilities on the ball throwing test. Adjusting for various features concerning the mother showed that this was not an independent association.

It is important to stress that these analyses have considered only those markers of the environment that were apparent up until the child reached the age of 4 weeks. The mechanisms by which the children’s ball throwing accuracy improves is likely to include the amount of practice and encouragement they receive from parents, friends, and relations and at preschool and infant school. These analyses were undertaken with the aim of assessing underlying features of the background that may be of importance. Not only have we emphasized the basic features of sex and gestation, but raised the issue of maternal stress in mid-childhood.

### Strengths and Limitations

There are a number of strengths to this study: (*a*) the numbers of children are large and the ball throwing test was undertaken under standardized conditions; (*b*) information analyzed was collected prior to the child being born, and hence was unlikely to be biased in regard to the motor ability of the child; (*c*) the data were able to be tested using the hypotheses generated from a review of the literature; none of the prenatal items tested were shown to be associated; (*d*) the hypothesis-free associations have identified a possible effect of the mother’s childhood stress on the child’s ability.

The limitations of the study lie in the following: (*a*) we do not have a different replication sample with similar data with which to test the maternal stress effect. However, the lack of a similar association with paternal childhood stress indicates the specificity of the maternal effect. It is also noteworthy that the negative associations were found with various indicators of stress including both physical and emotional abuse, staying in a Children’s Home and the mother’s perception of her mother as uncaring and unstable. These were all subsumed in a rating of maternal happiness in midchildhood that was positively associated with ball skills. (*b*) An obvious limitation concerns the choice of variables collected by the Avon Longitudinal Study of Parents and Children. Although they were chosen after detailed consideration of environmental features suggested in the literature to possibly have adverse effects on the fetus or child, they could not be definitive. In particular, there are many pollutants that can only be detected in biological assays and have not yet been tested in this sample. (*c*) Third, our emphasis on controlling the type II error rate may have led to a higher rate of false positives than is usually expected. Thus, several of the factors significant at the 5% level in Table 4 are likely to be due to chance.

In conclusion, we have shown that apart from sex and preterm associations, the markers in the literature indicating impaired motor ability showed little demonstrable effect on ball skills. Hypothesis-free associations, however, demonstrated features that may be of importance in understanding the etiology of throwing accuracy. These include a history of maternal prenatal abuse/stress, and current poor vision in the parents (particularly the father). Other associated factors may or may not be due to chance including the presence of dogs as pests in the neighborhood, maternal dietary features including frying with solid fat, or consumption of potato chips, or the frequency with which she has dieted. Further study of this cohort is expected to identify possible biological mechanisms by which the mother’s childhood experience might influence her child’s motor ability.

## Supplementary Material

Supplementary material
