# Supplementary material for: Parental, Prenatal, and Neonatal Associations With Ball Skills at Age 8 Using an Exposome Approach
Source: J Child Neurol. 2014 May 14;29(10):1390–8. doi: 10.1177/0883073814530501 (PMC4388909; doi:10.1177/0883073814530501)
Supplement: Supplementary material [file Supplementary_Tables.pdf]

Supplementary Table 1 Backwards step-wise regression of ball skills score measured at age 7

A. Mother's childhood

	Univariable			Intra domain		
**Variable**	**N**	**P**	**b [95% CI]**	**N**	**P**	**b [95% CI]**
Happiness in mid childhood: 6-11 (d761)	6544	<0.0001****	0.16 [0.09, 0.23]	6456	<0.0001****	0.16 [0.09, 0.23]
Happiness in late childhood: 12-15 (d762)	6593	<0.0001****	0.10 [0.05, 0.15]	6447	0.248	0.04 [-0.03, 0.11]
Step-sister present: 12-15 (d443)	6654	0.003**	0.62 [0.21, 1.04]	6456	<0.001***	0.77 [0.34, 1.20]
Maternal care score (d725)	6654	<0.001***	0.09 [0.03, 0.14]	6456	0.659	0.01 [-0.05, 0.08]
Stayed in a children's home (d402a)	6556	0.006**	-0.72 [-1.22, -0.21]	6456	0.018*	-0.64 [-1.16, -0.11]
Own mother stable & predictable (d750)	6483	0.008**	0.12 [0.03, 0.20]	6334	0.527	0.03 [-0.07, 0.13]
Home stability in childhood (d755)	6590	0.003**	0.10 [0.03, 0.17]	6429	0.582	0.02 [-0.06, 0.10]
Physically abused by parent (c416a)	6622	0.001**	-0.47 [-0.76, -0.18]	6289	0.324	-0.16 [-0.47, 0.16]
Emotionally abused by parent (c420a)	6622	<0.001***	-0.32 [-0.51, -0.13]	6289	0.369	-0.10 [-0.31, 0.12]

Total N=6456, Overall R^2=0.61%

Supplementary Table 1 Backwards step-wise regression of ball skills score measured at age 7

B. The mother's home environment

	Univariable			Intra domain		
**Variable**	**N**	**P**	**b [95% CI]**	**N**	**P**	**b [95% CI]**
Time lived in Avon (a002)	6715	<0.001***	0.07 [0.03, 0.11]	6239	0.082	0.04 [0.00, 0.08]
Number of moves in last 5 years (a005)	6574	0.004**	-0.07 [-0.12, -0.02]	6110	0.067	-0.05 [-0.10, 0.00]
Freq. windows open on summer day (a055)	6700	<0.001***	0.15 [0.07, 0.23]	6241	0.002**	0.13 [0.05, 0.22]
Cat(s) in the home (a062)	6720	0.010**	-0.14 [-0.25, -0.03]	6241	0.003**	-0.17 [-0.28, -0.05]
Dog(s) as pests in area (a076)	6720	0.007**	-0.12 [-0.20, -0.03]	6241	0.004**	-0.13 [-0.22, -0.04]
Level of damp/condensation in home (a081)	6668	0.007**	-0.12 [-0.20, -0.03]	6241	0.025*	-0.10 [-0.19, -0.01]
Number of areas wallpapered in last yr (a117)	6720	0.001**	0.08 [0.03, 0.13]	6241	0.011*	0.06 [0.01, 0.11]
Frequency of aerosol use (a141)	6745	<0.001***	0.06 [0.03, 0.09]	6241	0.006**	0.05 [0.01, 0.09]
Frequency of electric iron use (a323)	6398	<0.001***	0.10 [0.04, 0.16]	6241	0.239	0.04 [-0.03, 0.10]
Frequency of electric hair appliance use (a324)	6398	<0.0001****	0.08 [0.04, 0.12]	6241	0.010**	0.06 [0.01, 0.10]
Number of children in household (a502)	6781	0.007**	0.08 [0.02, 0.14]	6241	<0.001***	0.12 [0.05, 0.18]
Neighborhood is polluted/dirty (a635)	6617	0.003**	-0.14 [-0.23, -0.05]	6139	0.146	-0.07 [-0.16, 0.02]

Total N=6241, Overall R^2=1.20%

Supplementary Table 1 Backwards step-wise regression of ball skills score measured at age 7

C. Social and health

	Univariable			Intra domain		
**Variable**	**N**	**P**	**b [95% CI]**	**N**	**P**	**b [95% CI]**
Had a head injury (d120)	6568	0.009**	0.17 [0.04, 0.30]	5566	0.017*	0.17 [0.03, 0.31]
Poor vision (d263)	6654	0.003**	-0.33 [-0.54, -0.11]	5566	0.010**	-0.30 [-0.54, -0.07]
Partner had children not living at home (a534)	6781	0.009**	0.24 [0.06, 0.42]	5566	0.013*	0.25 [0.05, 0.45]
Mother's educational qualifications (c645a)	6647	0.004**	-0.06 [-0.10, -0.02]	5554	0.142	-0.04 [-0.08, 0.01]
Length of time on contraceptive pill (d022)	6281	0.002**	0.08 [0.03, 0.14]	5566	0.010**	0.08 [0.02, 0.13]
Number of living children (b005)	6617	0.008**	0.08 [0.02, 0.14]	5462	0.179	0.04 [-0.02, 0.11]
Diarrhea in third trimester (c054)	6498	0.008**	0.15 [0.04, 0.26]	5448	0.071	0.11 [-0.01, 0.23]
X-ray in last months of pregnancy (e114)	6482	0.008**	-0.42 [-0.73, -0.11]	5360	0.083	-0.30 [-0.64, 0.04]
X-ray of leg/foot in pregnancy (d308)	6654	0.001**	-1.10 [-1.77, -0.42]	5566	0.071	-0.75 [-1.57, 0.07]
Worry about home being burgled (a620)	6544	0.007**	-0.09 [-0.16, -0.02]	5566	0.007**	-0.10 [-0.17, -0.03]
Mother was Church of England (d813_ce)	6542	<0.001***	0.19 [0.08, 0.29]	5566	0.009**	0.15 [0.04, 0.26]
Freq. mother attends place of worship (d816)	6495	0.003**	-0.08 [-0.14, -0.03]	5566	0.024*	-0.07 [-0.13, -0.01]
Mother has help from others in religion (d818)	6276	0.005**	-0.24 [-0.40, -0.07]	5364	0.720	-0.04 [-0.28, 0.19]
Feels discriminated against b/c of dress (c792)	6503	0.004**	-0.46 [-0.77, -0.15]	5566	0.018*	-0.43 [-0.78, -0.07]

Total N=5566, Overall R^2=0.97%

Supplementary Table 1 Backwards step-wise regression of ball skills score measured at age 7

D. Mother's diet

	Univariable			Intra domain		
**Variable**	**N**	**P**	**b [95% CI]**	**N**	**P**	**b [95% CI]**
Has milk in tea during pregnancy (c284)	6501	0.001**	0.12 [0.05, 0.19]	6241	0.005**	0.10 [0.03, 0.17]
Freq. of eating poultry in pregnancy (c203)	6492	0.002**	0.11 [0.04, 0.18]	6241	0.007**	0.10 [0.03, 0.17]
Freq. of eating pulses in pregnancy (c240)	6497	0.001**	-0.12 [-0.19, -0.05]	6240	0.248	-0.05 [-0.13, 0.03]
Butter/lard/fat used for frying in preg. (c261)	6475	0.002**	0.19 [0.07, 0.32]	6241	0.010**	0.17 [0.04, 0.30]
Freq. of eating potato crisps in preg. (c219)	6492	<0.001***	0.08 [0.04, 0.13]	6241	0.005**	0.07 [0.02, 0.12]
Number of times mother has dieted (c336)	6543	0.006**	0.06 [0.02, 0.11]	6241	0.006**	0.07 [0.02, 0.11]
Has ever been a vegetarian (c340)	6557	0.005**	-0.14 [-0.23, -0.04]	6154	0.371	-0.05 [-0.17, 0.06]
Freq. drank herbal tea in 3rd trimester (c315)	6477	<0.001***	-0.18 [-0.27, -0.09]	6241	0.006**	-0.14 [-0.23, -0.04]
Frequency of binge drinking mid-preg. (b723)	6720	0.008**	0.10 [0.03, 0.17]	6241	0.007**	0.10 [0.03, 0.18]

Total N=6241, Overall R^2=0.98%

Supplementary Table 1 Backwards step-wise regression of ball skills score measured at age 7

E. The neonate (excluding obstetric variables)

	Univariable			Intra domain		
**Variable**	**N**	**P**	**b [95% CI]**	**N**	**P**	**b [95% CI]**
Sex: girl v boy (kz021)	7329	<0.0001****	-0.73 [-0.83, -0.64]	6867	<0.0001****	-0.74 [-0.84, -0.64]
Birthweight (kz030)	6867	<0.0001****	0.14 [0.09, 0.19]	6867	0.033*	0.06 [0.01, 0.12]
Head circumference (kz031c)	4727	<0.0001****	0.12 [0.06, 0.18]	4696	0.295	-0.05 [-0.13, 0.04]
Birth length (kz032c)	4648	<0.0001****	0.14 [0.08, 0.20]	4618	0.589	0.03 [-0.08, 0.14]
Gestation at delivery[a] (kz029a)	6913	<0.0001****	0.40 [0.21, 0.58]	6867	0.003**	0.32 [0.11, 0.53]

Total N=6867, Overall R^2=3.65%

Supplementary Table 1 Backwards step-wise regression of ball skills score measured at age 7

F. The neonate (including obstetric variables)

	Univariable			Intra domain		
**Variable**	**N**	**P**	**b [95% CI]**	**N**	**P**	**b [95% CI]**
Sex: girl v boy (kz021)	7329	<0.0001****	-0.73 [-0.83, -0.64]	4016	<0.0001****	-0.76 [-0.89, -0.63]
Birthweight (kz030)	6867	<0.0001****	0.14 [0.09, 0.19]	4016	0.036*	0.08 [0.01, 0.16]
Head circumference (kz031c)	4727	<0.0001****	0.12 [0.06, 0.18]	2836	0.227	-0.07 [-0.19, 0.04]
Birth length (kz032c)	4648	<0.0001****	0.14 [0.08, 0.20]	2789	0.593	0.04 [-0.10, 0.18]
Gestation at delivery[a] (kz029a)	6913	<0.0001****	0.40 [0.21, 0.58]	4016	0.011*	0.34 [0.08, 0.61]
Baby was resuscitated (del_b4005)	4038	0.005**	-0.20 [-0.34, -0.06]	4016	0.015*	-0.18 [-0.32, -0.03]
Resus. with intubation and IPPV (del_b4011)	4038	0.007**	-0.56 [-0.97, -0.15]	4016	0.173	-0.29 [-0.71, 0.13]
Baby transferred (del_b4050)	4048	0.001**	-0.19 [-0.31, -0.07]	4015	0.667	-0.03 [-0.18, 0.11]

Total N=4016, Overall R^2=4.13%

[a] coded in 3 categories: <32, 32-36, 37+ weeks

Supplementary Table 1 Backwards step-wise regression of ball skills score measured at age 7

G. The father – Analysis 1

	Univariable			Intra domain		
**Variable**	**N**	**P**	**b [95% CI]**	**N**	**P**	**b [95% CI]**
Freq. of eating meat pies in pregnancy (pb021)	5386	0.007**	0.10 [0.03, 0.17]	4933	0.030*	0.08 [0.01, 0.16]
Father is vegetarian/vegan (pb069)	5385	0.009**	-0.38 [-0.67, -0.09]	4933	0.043*	-0.33 [-0.64, -0.01]
Fragile inner self (pb550)	5283	0.003**	-0.09 [-0.14, -0.03]	4933	0.009**	-0.08 [-0.14, -0.02]
Interpersonal sensitivity score (pb551)	5295	0.010**	-0.08 [-0.14, -0.02]	4933	0.553	-0.03 [-0.11, 0.06]
Freq. of attending place of worship (pb155)	5266	0.009**	-0.09 [-0.15, -0.02]	4917	0.419	-0.04 [-0.12, 0.05]
Obtains help from leader of religion (pb156)	5105	0.007**	-0.31 [-0.54, -0.08]	4933	0.014*	-0.29 [-0.52, -0.06]
Helped by other members of religion (pb157)	5060	0.003**	-0.32 [-0.53, -0.10]	4864	0.413	-0.15 [-0.50, 0.21]

Total N=4933, Overall R^2=0.52%

Supplementary Table 1 Backwards step-wise regression of ball skills score measured at age 7

H. The father – Analysis 2

	Univariable			Intra domain		
**Variable**	**N**	**P**	**b [95% CI]**	**N**	**P**	**b [95% CI]**
Sight in left eye without glasses (pa270)	4765	<0.001***	-0.13 [-0.20, -0.06]	4591	0.601	-0.03 [-0.15, 0.09]
Sight in right eye without glasses (pa271)	4751	0.004**	-0.11 [-0.18, -0.03]	4591	0.494	0.04 [-0.08, 0.16]
Any sight problem (pa272)	4732	<0.0001****	-0.25 [-0.38, -0.13]	4591	<0.001***	-0.23 [-0.35, -0.10]
Time lived in Avon (pa002)	4824	0.005**	0.07 [0.02, 0.12]	4581	0.070	0.05 [0.00, 0.10]
Bedrooms' low temperature in winter (pa007)	4793	0.004**	-0.11 [-0.19, -0.04]	4591	0.034*	-0.09 [-0.17, -0.01]
Deodorant used (pa043)	4862	0.002**	0.09 [0.04, 0.15]	4591	0.008**	0.08 [0.02, 0.14]
Use of ionizing radiation (pa053)	4846	0.005**	-0.24 [-0.41, -0.07]	4591	0.009**	-0.23 [-0.40, -0.06]
Anxious about being burgled (pa020)	4778	0.006**	-0.11 [-0.19, -0.03]	4591	0.032*	-0.09 [-0.17, -0.01]

Total N=4591, Overall R^2=0.88%

Supplementary Table 1   Backwards step-wise regression of ball skills score measured at age 7

I. Significant variables from analyses G and H (The father)

	Univariable			Intra domain		
**Variable**	**N**	**P**	**b [95% CI]**	**N**	**P**	**b [95% CI]**
Freq. of eating meat pies etc (pb021)	5386	0.007**	0.10 [0.03, 0.17]	4339	0.010*	0.10 [0.02, 0.18]
Father is vegetarian/vegan (pb069)	5385	0.009**	-0.38 [-0.67, -0.09]	4309	0.102	-0.27 [-0.60, 0.05]
Fragile inner self (pb550)	5283	0.003**	-0.09 [-0.14, -0.03]	4339	0.024*	-0.07 [-0.14, -0.01]
Obtains help from leader of religion (pb156)	5105	0.007**	-0.31 [-0.54, -0.08]	4121	0.075	-0.22 [-0.47, 0.02]
Any sight problem (pa272)	4732	<0.0001****	-0.25 [-0.38, -0.13]	4339	<0.001***	-0.22 [-0.34, -0.09]
Bedrooms’ low temperature in winter (pa007)	4793	0.004**	-0.11 [-0.19, -0.04]	4301	0.053	-0.08 [-0.17, 0.00]
Deodorant used (pa043)	4862	0.002**	0.09 [0.04, 0.15]	4339	0.024*	0.07 [0.01, 0.13]
Use of radiation (pa053)	4846	0.005**	-0.24 [-0.41, -0.07]	4339	0.010*	-0.22 [-0.40, -0.05]
Anxious about being burgled (pa020)	4778	0.006**	-0.11 [-0.19, -0.03]	4293	0.077	-0.08 [-0.16, 0.01]

Total N=4339, Overall R^2=0.95%

Supplementary Table 2 Backwards stepwise regression of ball skills score measured at age 7 years combining all data significant in earlier analyses.

Final model

	Univariable			Fully adjusted		
**Variable**	**N**	**P**	**b [95% CI]**	**N**	**P**	**b [95% CI]**
Study father						
Freq. of eating meat pies in pregnancy (pb021)	5386	0.007**	0.10 [0.03, 0.17]	4017	0.028*	0.09 [0.01, 0.17]
Fragile inner self (pb550)	5283	0.003**	-0.09 [-0.14, -0.03]	3952	0.320	-0.03 [-0.10, 0.03]
Any sight problem (pa272)	4732	<0.0001****	-0.25 [-0.38, -0.13]	4017	0.004**	-0.19 [-0.31, -0.06]
Deodorant used (pa043)	4862	0.002**	0.09 [0.04, 0.15]	4010	0.085	0.06 [-0.01, 0.12]
Use of radiation (pa053)	4846	0.005**	-0.24 [-0.41, -0.07]	4017	0.039*	-0.18 [-0.35, -0.01]
The neonate						
Sex: girl v boy (kz021)	7329	<0.0001****	-0.73 [-0.83, -0.64]	4017	<0.0001****	-0.76 [-0.88, -0.63]
Gestation at delivery[a] (kz029a)	6913	<0.0001****	0.40 [0.21, 0.58]	4017	0.006**	0.36 [0.10, 0.62]
The mother						
Happiness in mid childhood: 6-11 (d761)	6544	<0.0001****	0.16 [0.09, 0.23]	4017	<0.001***	0.15 [0.07, 0.24]
Step-sister present: 12-15 (d443)	6654	0.003**	0.62 [0.21, 1.04]	4017	0.010**	0.74 [0.18, 1.30]
Freq. of eating poultry in pregnancy (c203)	6492	0.002**	0.11 [0.04, 0.18]	4017	0.157	0.06 [-0.02, 0.15]
Butter/lard/fat used for frying in preg. (c261)	6475	0.002**	0.19 [0.07, 0.32]	4017	0.009**	0.21 [0.05, 0.37]
Freq. of eating potato crisps in preg. (c219)	6492	<0.001***	0.08 [0.04, 0.13]	4017	0.023*	0.07 [0.01, 0.13]
Number of times mother has dieted (c336)	6543	0.006**	0.06 [0.02, 0.11]	4017	0.008**	0.08 [0.02, 0.13]
Freq. windows open on summer day (a055)	6700	<0.001***	0.15 [0.07, 0.23]	4017	0.001**	0.17 [0.07, 0.27]

Cats in the home (a062)	6720	0.010**	-0.14 [-0.25, -0.03]	4017	0.016*	-0.16 [-0.30, -0.03]
Dogs as pests (a076)	6720	0.007**	-0.12 [-0.20, -0.03]	4017	<0.001***	-0.18 [-0.29, -0.08]
Level of damp/condensation in home (a081)	6668	0.007**	-0.12 [-0.20, -0.03]	4017	0.041*	-0.11 [-0.22, 0.00]
Number of areas wallpapered in last yr (a117)	6720	0.001**	0.08 [0.03, 0.13]	4017	0.176	0.04 [-0.02, 0.10]
Frequency of electric hair appliance use (a324)	6398	<0.0001****	0.08 [0.04, 0.12]	3901	0.272	0.03 [-0.02, 0.08]
Number of children in household (a502)	6781	0.007**	0.08 [0.02, 0.14]	4017	0.070	0.07 [-0.01, 0.15]
Had a head injury (d120)	6568	0.009**	0.17 [0.04, 0.30]	3979	0.284	0.09 [-0.07, 0.25]
Poor vision (d263)	6654	0.003**	-0.33 [-0.54, -0.11]	4017	0.257	-0.15 [-0.40, 0.11]
Freq. mother attends place of worship (d816)	6495	0.003**	-0.08 [-0.14, -0.03]	4017	0.003**	-0.10 [-0.17, -0.04]

Total N=4017, Overall R^2=5.90%

[a] coded in 3 categories: <32, 32-36, 37+ weeks

Supplementary Table 3   Final model combining features of the mother and the neonate: adjusted odds of offspring having poor ball skills

	Univariable			Intra domain		
**Variable**	**N**	**P**	**OR [95% CI]**	**N**	**P**	**OR [95% CI]**
Happiness in mid childhood: 6-11 (d761)	6544	<0.0001****	0.82 [0.76, 0.89]	5595	0.002**	0.86 [0.78, 0.95]
Had a head injury (d120)	6568	0.029*	0.81 [0.67, 0.98]	5595	0.008**	0.75 [0.61, 0.93]
Cats in the home (a062)	6720	0.001**	1.26 [1.09, 1.45]	5595	0.002**	1.29 [1.10, 1.51]
Dogs as pests (a076)	6720	0.017*	1.14 [1.02, 1.27]	5595	0.001**	1.23 [1.09, 1.39]
Number of areas wallpapered in last yr (a117)	6720	0.018*	0.92 [0.86, 0.99]	5595	0.036*	0.92 [0.85, 0.99]
Frequency of electric iron use (a323)	6398	<0.0001****	0.84 [0.78, 0.92]	5595	0.001**	0.86 [0.79, 0.94]
Worry about home being burgled (a620)	6544	<0.001***	1.17 [1.06, 1.28]	5595	0.043*	1.11 [1.00, 1.23]
Partner had children not living at home (a534)	6781	0.007**	0.68 [0.52, 0.90]	5595	0.011*	0.66 [0.48, 0.91]
Freq. of eating potato crisps in preg. (c219)	6492	0.002**	0.90 [0.85, 0.96]	5595	0.009**	0.91 [0.84, 0.98]
Mother was Church of England (d813_ce)	6542	0.001**	0.80 [0.69, 0.92]	5595	0.031*	0.84 [0.72, 0.98]
Feels discriminated against b/c of dress (c792)	6503	<0.001***	1.91 [1.34, 2.71]	5595	0.046*	1.55 [1.01, 2.38]
Gestation at delivery (kz029a)	6913	0.009**	0.80 [0.68, 0.95]	5595	0.022*	0.79 [0.64, 0.97]
Sex: girl v boy (kz021)	7329	<0.0001****	1.77 [1.54, 2.02]	5595	<0.0001****	1.74 [1.49, 2.03]

Total N=5595, Overall pseudo R^2=3.13%
